# Scientific Investigations of the Cranium and Jaws

**Published:** 1891-05

**Authors:** Eugene S. Talbot


### THE DENTAL REGISTER.

VOL. XLV.] MAY, 1891. [No. 5.

### COMMUNICATIONS.

### Scientific Investigations of the Cranium and Jaws.

BY EUGENE S. TALBOT, M.D., D.D.S.

Read before the Mississippi Valley Dental Association, March 11, 1891.

In a hurried manner I have sketched a few ideas as they have occurred to me in regard to the forthcoming investigation of the jaws of modern and prehistoric skulls. At the last meeting of the American Dental Association, Dr. J. J. R. Patrick of Belleville, Ill., was appointed to take direct surpervision of the work of the examination of the jaws and teeth of the skulls in the different museums of this country. The Association could not have selected a better man. It is quite essential that some one who has had years of practical training like Dr. Patrick in this particular line of work should not only supervise but actually make the examination. From the experience which I have had in the past ten years, in making examination of the work for data that are not so particular or difficult, I have learned, that it is very essential that the work should be done by a man or a set of men of experience and that they should go through all of the museums and superintend personally all the work. These men should be men of experience and integrity whose names connected with any paper would be sufficient guarantee of the accuracy and completeness of the investigation.

I can not see how it is possible for even one individual to go on with the work until some clear and systematic plan has been devised as a foundation to work upon. This plan can not be successfully laid out by any one, or ten individuals, for the reason that no individual is able to grasp all the points that are liable to arise in a complete scientific research. This argument is illustrated by the remarks made by Dr. Patrick, showing that even he, with his large fund of knowledge and personal experience, is liable to be misled by some personal view in regard to this matter. Among other things he says on page 70 of the Transactions of the Illinois State Dental Society for 1890: "I received some cuts of the surperior maxilla, a full set of teeth, and the writer desired to know the measurment of some of my prehistoric skulls, that is, the transverse diameter of the dental arch in the region of the sixth year molars. So far as race characteristics are concerned there can be no object in such an investigation. And so with the depth of the palate, etc.; all these measurements are futile for a number of reasons which it would be occupying too much time to consider." Now, while these measurements may not be of any value so far as race characteristics are concerned, (which I will not admit), yet it is just as essential that a very accurate measurement should be made across the upper jaw in the region of the first permanent molar as any other examination of the mouth or jaw.

It is barely possible that Dr. Patrick has certain reasons for thinking that these measurements are not necessary and yet it is possible that some one might require these particular measurements in the near future and find upon examination of the report that they had not been taken, thus necessitating the work of going all over the ground again. This state of things has already occurred in the very laborious and exhaustive report of Dr. Betty. After Dr. Patrick has received suggestions from the dentists, he should compare them with his own views and then publish his chart in the leading dental journals at intervals, for some time, inviting criticisms in the journals from the dentists so that these criticisms may be read by all. In this way the views of those interested in the original investigations can be recorded. The suggestions that are of value can be added to the chart and such points as are not of material use can be discarded. In this manner a tolerable accurate chart may be produced.

All the measurements across the superior maxilla up to the time of Dr. Betty's examination, were made from first molar to first molar. What motive could have induced Dr. Betty to make a change and meausure from third molar to third molar I can not understand, although he may possess good and sufficient reasons for so doing. I shall have occasion to use the measurement from first molar to first molar in a paper I have been collecting material for, during the past ten years. I congratulate myself that a large expenditure of time and money was saved by the work already done by Dr. Betty. I now find that unless he can show that his plan of measurement is the better one, I must go all over the work again thus causing considerable annoyance to the officers of the museum. Years ago the late Dr. John Mummery recognized the fact that the only parts giving a true and accurate development of the jaw was at the anterior root of the first permanent molar and this is very natural. The first permanent molars are the only teeth that erupt normally and are not influenced by the other teeth. They are the teeth by which most of the work of mastication is accomplished and therefore the jaw is better developed at that point; the roots of the teeth give a very accurate point by which to obtain the diameter of the jaw, while on the other hand the third molars never erupt normally, they are creatures of circumstances, sometimes erupting outside of the alveolar process, sometimes inside, again in the centre of the process, being always at the mercy of the second molar teeth.

The third molar always erupts after the jaw has developed while the first permanent molar erupts before the development of the alveolar process and is carried undisturbed laterally with it. Again, the alveolar process at the extremities is always influenced by the surrounding parts, the divergence of the wisdom on the lower jaw, the tongue and the cheeks, while the first permanent molars are always held in position by each other. Frequently one wisdom tooth or both are missing and in that case it would be impossible to get the measurement. Some one will say that this is the case with the first permanent molar; so it is, but the second molar in that case will answer the purpose if it moves forward and fills the space made vacant by the extraction of the first permanent molar, when a nearly accurate measurement is as readily obtained; the buccal roots of this tooth giving the precise diameter of the jaw at the time the measurement is taken. The diameter of the jaw at the location of the second molar, when all are in place, is greater than the diameter at the first permanent molar, so that if the first molar should be removed the second molar coming forward will occupy about the same position in the jaw that the first molar did.

I consider the antero-posterior diameter of as much importance as the lateral diameter provided a large collection can be made so as to obtain the average excessive and arrested development, and the premature extraction of teeth goes far toward interfering with the true character of the jaw. The forward movement of the teeth as a result of the above mentioned causes is bound to effect a true solution of race characteristics. Prognathism of either the upper or lower jaw frequently results from abnormal development of the opposite jaw, resulting in a local cause. Again the constant extraction of the first permanent molar or want of eruption of the third molar would preclude any attempt on the part of the investigator to make an accurate investigation.

As Dr. Owen is able to classify some of the lower animals by the antero-posterior diameter of the jaw, so may we with proper measurement, be able to add materially to our knowledge of races by this means. Dr. Patrick also says on same page: "With regard to measurements of human crania. This has puzzled the brain and ingenuity of physiognomists and craniologists for the last 150 years. They have arrived at no reliable data in regard to the relative size of the cranium and face bones. Measurements of crania amount to comparatively nothing as designating brain capacity or race."

I quite agree with the doctor so far as the measurements of the cranium are concerned, but should we, as dentists, (specialists) who are supposed to be familiar with the bones of the cranium, become so narrow in our investigations as to confine ourselves in a scientific study of so much importance as this is, to nothing but the teeth? I had always supposed from his writings that Dr.

Patrick was a broad-gauged scientific investigator, not only in craniology but also in osteology, and that he, above all others, with his vast knowledge of craniology, would for his own benefit, if not for the general public, wish to explore the whole field and show to all scientists that this commission appointed by the representative body of dentists, was able to place new facts and new data before the world so that physiognomists and craniologists the world over can look upon the report of the committee as not only final, but display such a fund of information that the scholars of all countries will look upon it as a piece of masterly work. That physiognomists and craniologists have not made headway in the past 150 years is true. And why have they not made headway? Simply because the starting points for the measurements were not accurate to commence with. For instance, take the facial angle.

Different authors possessed different notions in regard to what the facial angle should consist of. In Camper's angle the facial line is drawn between the most prominent points of the forehead and lower face, the basal line from the center of the external auditory meatus through the anterior nasal spine to meet the first. In Cuvier's angle, the two lines start from the same point to meet at the tip of the incisors; in Cloquet's, at the center of the superior alveolar border; in Jacquart's, at the anterior nasal spine.

The Munich-Frankfort angle (adopted at the craniometrical congress at Frankfort) takes the facial line between the supraciliary depression and the most prominent part of the alveolar border of the superior maxillary; the basal line is the so-called horizontal line drawn between the upper border of the external auditory meatus and the lower border of the orbit.

Topinard's angle has a facial line drawn from the supraciliary depression to the most prominent part of the lower face, and a basal line in the plane tangent to the occipital condyles and the upper alveolar arch. It will be noticed that most of these authors take for their starting point the external auditory meatus, drawing a line to the nasal spine, and yet all are at variance in regard to what the other points shall be. It seems never to have occurred to these men that the bones of the body never develop in harmony with the opposite sides or that the bones of one individual may not develop like those of another individual. I have seen people with one ear higher than the other—others with eyes higher or lower in the head than normal, or one eye higher in the head than the other; others have jaws either anterior or posterior to the normal position. The same is also true of the nasal spine. We frequently observe the nasal bone projecting or receding owing to the period of development and ossification of the bones at the base of the brain. How frequently we see excessive development and a want of development of the supraciliary ridges, as well as the anterior alveolar processes. A line extending two to four inches from any one of these points would necessarially make a vast difference in the shape of the calvarium.

It is a singular fact that less is known of the development of the osseous system than any other part of the body. In my investigations upon this subject I had occasion to go through the Army Medical Library at Washington and the only mention of this subject in any language consisted of twenty-four cases of excessive or arrested development, in connection with patients who were otherwise deformed; these were only mentioned in a general way. A small work upon this subject has been published lately by Dr. Sutton, an English author. It is very elementary although exceedingly interesting.

Dr. Patrick also says: "I care not what race of people you might come in contact with, however isolated in any island in the ocean, you could not find eight crania but would be different in shape and size from each other." In this statement, Dr. Patrick is quite correct.

One of the most complete, scientific and well-classified museums in Europe is that located at Wiesbaden, Germany; each room is numbered according to a special period in the world's history and anything of interest that could be obtained in relation to that particular period has been placed in that room in the natural position in which they were found. The first room contains relics pertaining to the Glacial period and bronze ages.

Among other things side by side are two skulls, one Brachycephalic, the other just the opposite, Dolichocephalic, thus showing that from the earliest man the shapes of the head differed.

Pages could be written about these two skulls alone. I have always observed that in the brachycephalic skull the upper jaw like the skull is always broad, as in the form of the jaw of the Chinese; while the dolichocephalic skull has a long, narrow jaw as observed in the negro. Notwithstanding all these differences in points of measurements, a good deal has been accomplished by craniologists. There is no class of men so capable of working out a new system of measurement of the skulls and establishing definite points as the dentists, and the present opportunity is one that seldom comes to a man whereby he can make a name for himself. It only requires thought, inventive skill and well-directed efforts to study out a plan which shall be superior to all others for the accurate measurements of the cranium and jaws.

Another point in Dr. Betty's paper. Dr. Black on page 75 of the Transactions of the Illinois State Dental Society, says: "I have not counted the irregularities in the individual skull, but in 150 Mound Builders there are 45 cases of irregularity or 30 per cent.; in 145 Indians, 16 cases of irregularity or 11 per cent." It is very essential for us to know whether these cases are the result of local causes or whether they are of constitutional origin. I have since gone over the tables of Dr. Betty and have found that only three can be considered constitutional—that is, irregularity resulting from arrest of development of the jaw. All the others are of local origin. I doubt if there is any other point in the whole investigation that is of more importance to us or to future investigators than the knowledge of excessive or arrested development of the jaws. We would expect to find more local irregularities of the teeth among early races than among the present people, because at that time the knowledge of the care of the teeth was limited, and the temporary teeth which were allowed to remain in the mouth until forced out by the second set, frequently caused the second set to rotate or to be forced out of their normal condition. Another point is as to whether a skull is that of a male or female. Dr. Patrick says: "There is no male or female skull." In this I think he intends to say that it is quite difficult to distinguish a male from a female skull. That there is quite a difference in size and shape every one will admit.

Let me ask what will all the investigation amount to if, after the committee finishes its work, it is unable to classify the skulls into male and female. The influences which produce decay of the teeth are not the same in the male and female. The jaws are smaller in the female than in the male. The diameters of the cranium, the weight of the brain, are known to be less in the female than in the male, and I should expect to find a difference in the shape of the jaws if a close inspection were made. It is known that the sutures of the female skull are more prominent than those of the male. The discovery of these points by some dentist whereby he can easily determine the sex will certainly add credit to his name.

